# Digitalis and Chloral in Delirium Tremens

**Published:** 1874-01

**Authors:** 


Digitalis and Chloral in Delirium Tremens.—Dr. E. Chenery relates a case of a Scotchman, aged thirty-five, suffering delirium tremens: "When I first saw him, he had neither taken food nor slept for nearly a week, and rejected everything which was put into his stomach; his mind was greatly agitated, and his whole muscular system was in a state of continual unrest. Pulse 120, and could not be counted at the wrist on account of the commotion among the tendons. He had taken bromide potassium, without effect, before my visit; a strong mustard plaster applied to pit of stomach, fifteen grains chloral given, and, in twenty minutes, twenty drops tincture digitalis, both retained and had favorable effect. Ten minutes after digitalis, dose of thirty grains chloral soon brought on sleep of three hours, when he woke with relief and better state of mind. Raw egg and milk given with another dose of digitalis, in a short time thirty grains of chloral. From this he passed into quiet sleep. Digitalis given three times daily for several days, partly to moderate the pulse, but mainly for its eliminating effect upon the kidneys; small doses of chloral as required. The cure, taken all in all, was the most satisfactory that could have been desired.

